# Supplementary material for: The pod components of the Shigella T3SS sorting platform accommodate multiple copies of Spa33 (SctQ)
Source: PLoS Pathog. 2026 Sep 21;22(9):e1014592. doi: 10.1371/journal.ppat.1014592 (PMC13614641; doi:10.1371/journal.ppat.1014592)
Supplement: Supplementary file 1 [file ppat.1014592.s002.pdf]

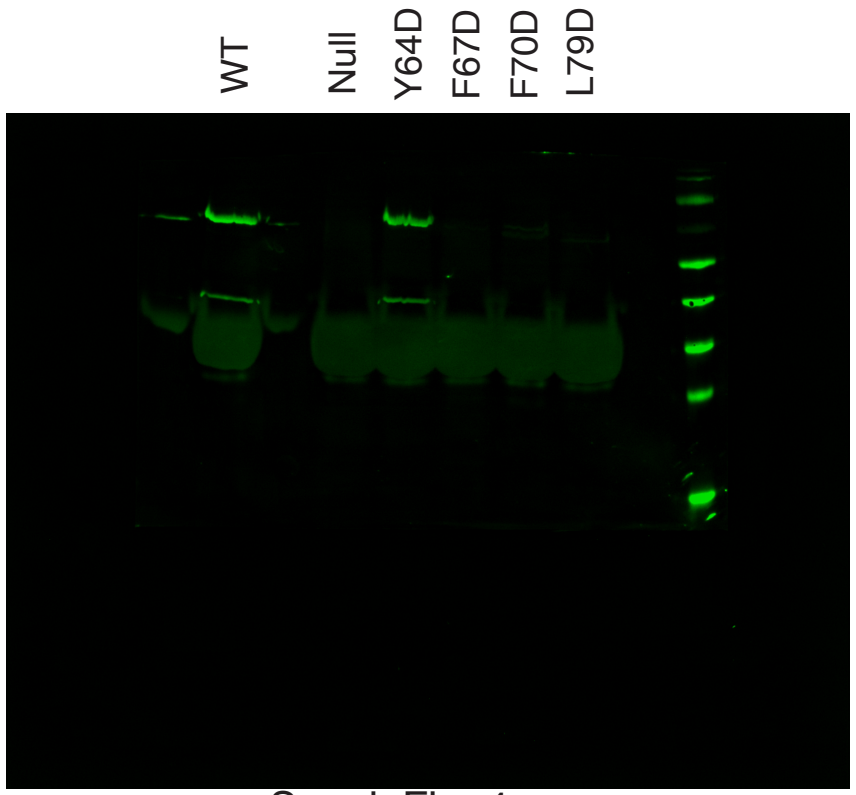

Suppl. Fig. 4a

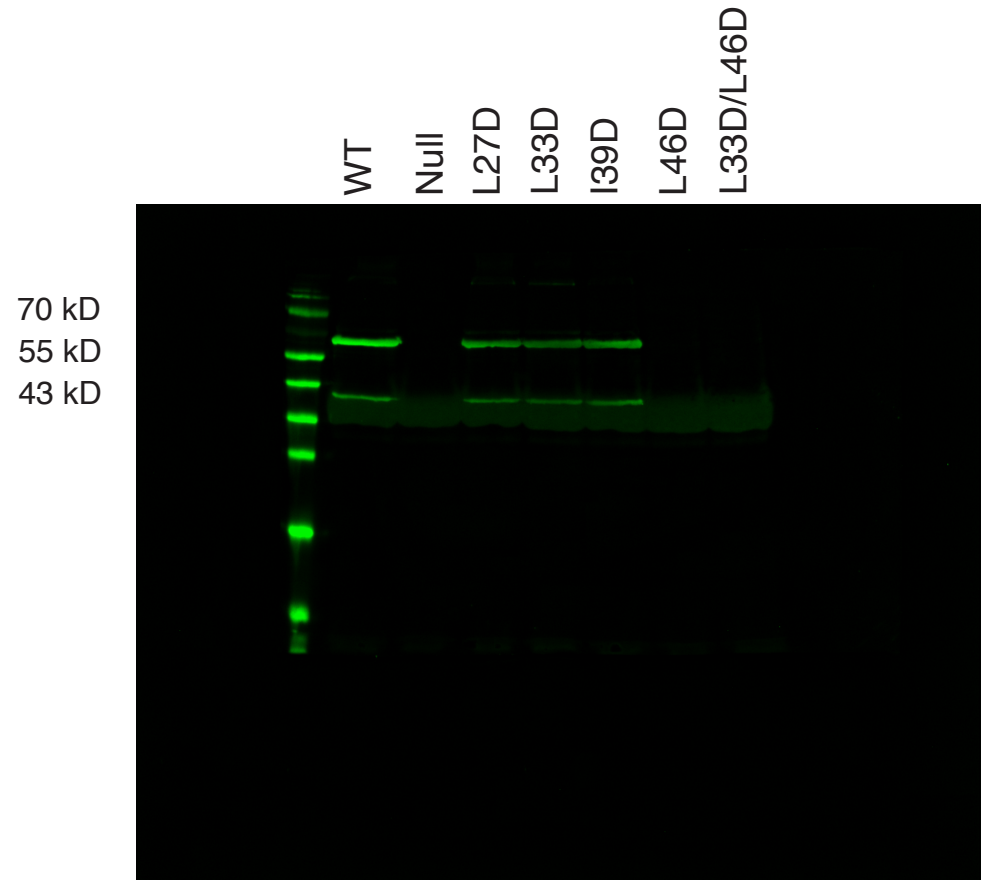

Suppl. Fig. 4b

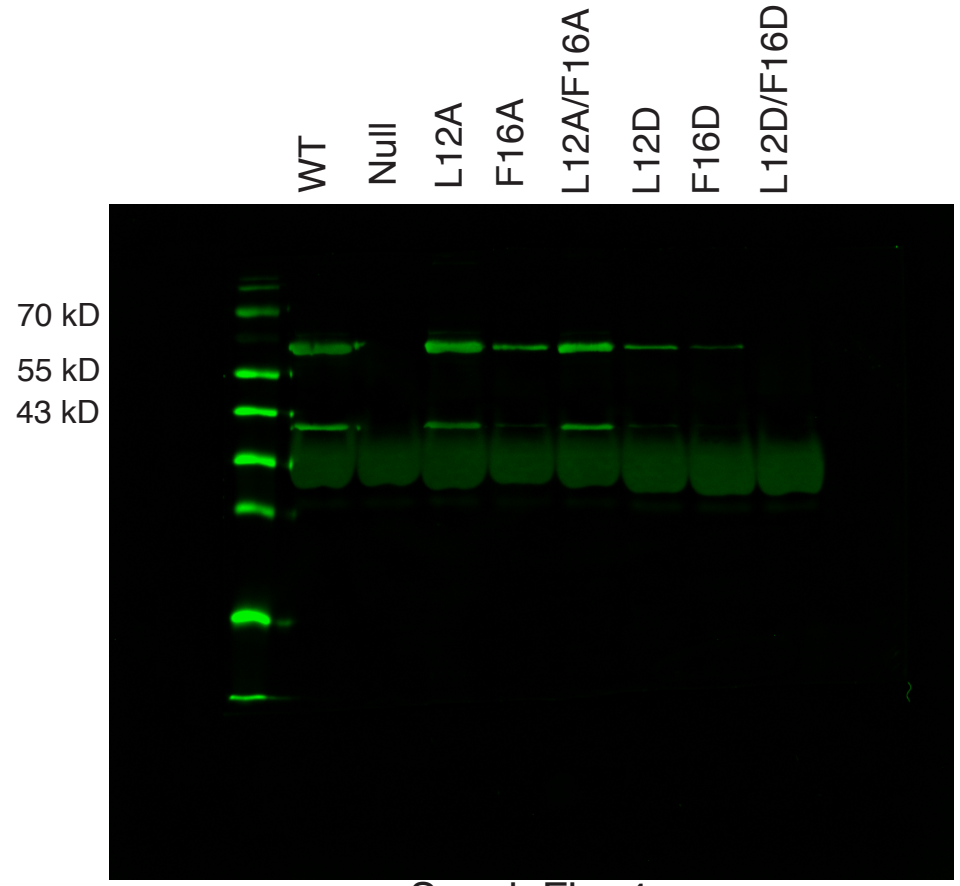

Suppl. Fig. 4c

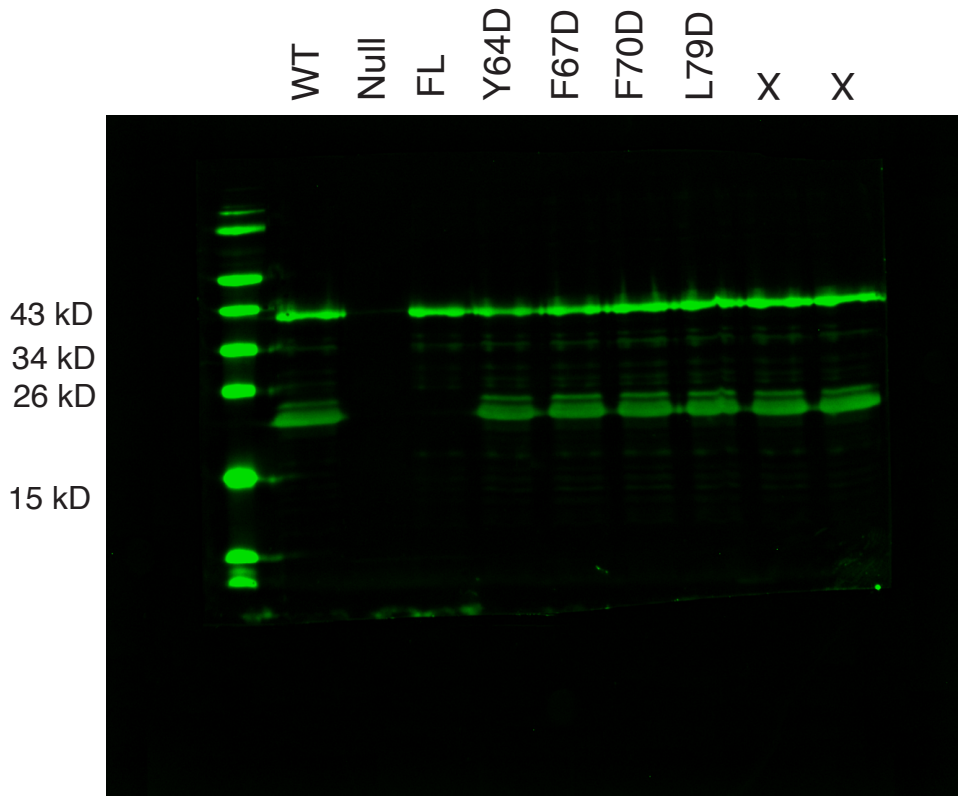

Suppl. Fig. 5a

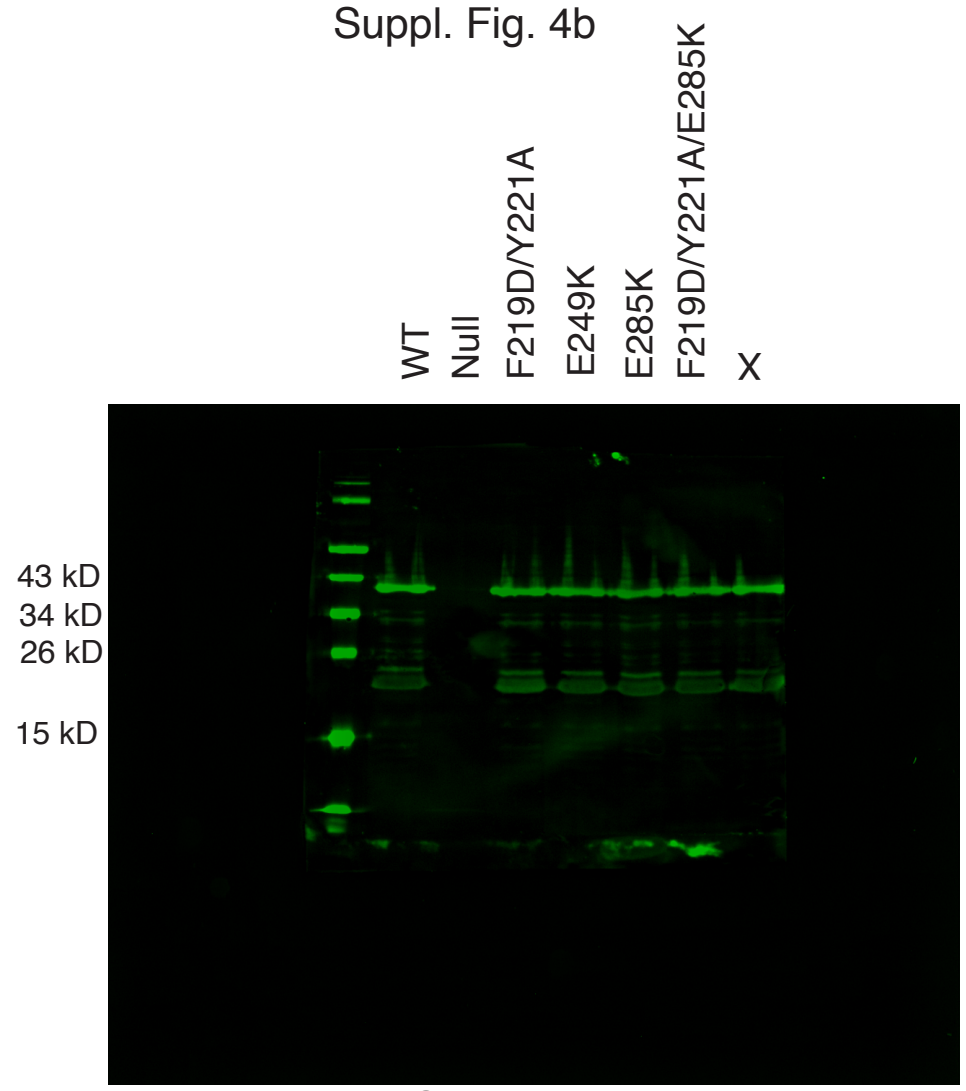

Suppl. Fig. 5b

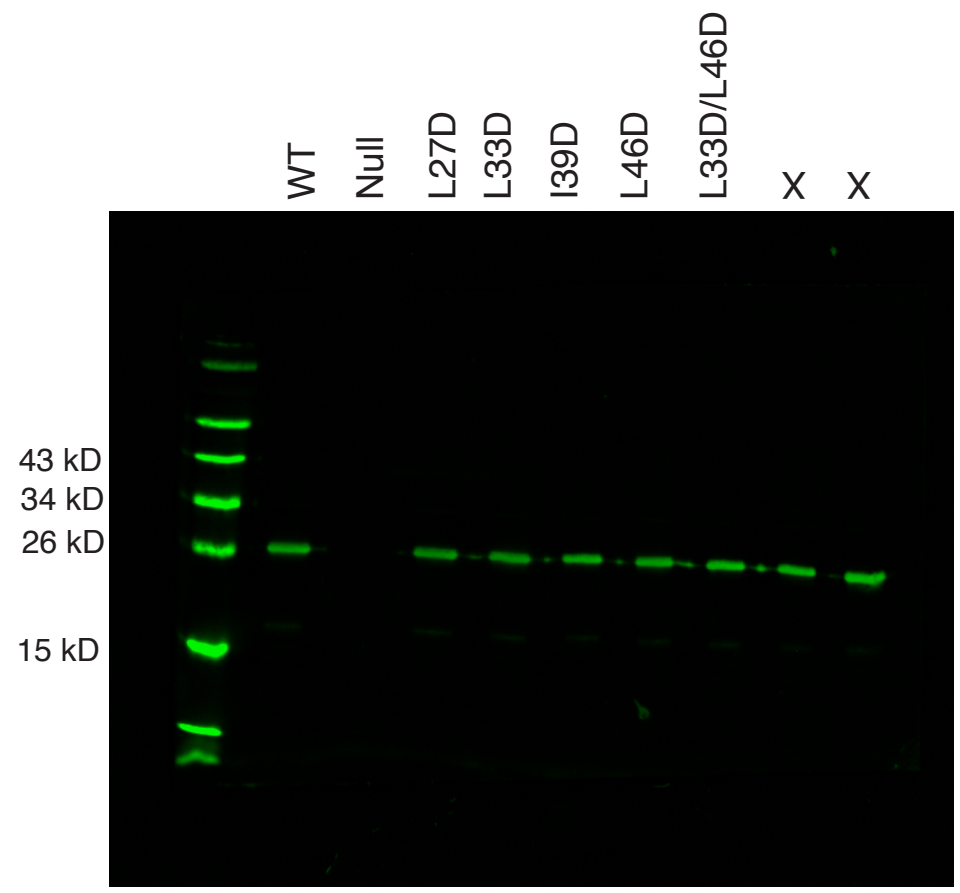

Suppl. Fig. 5c

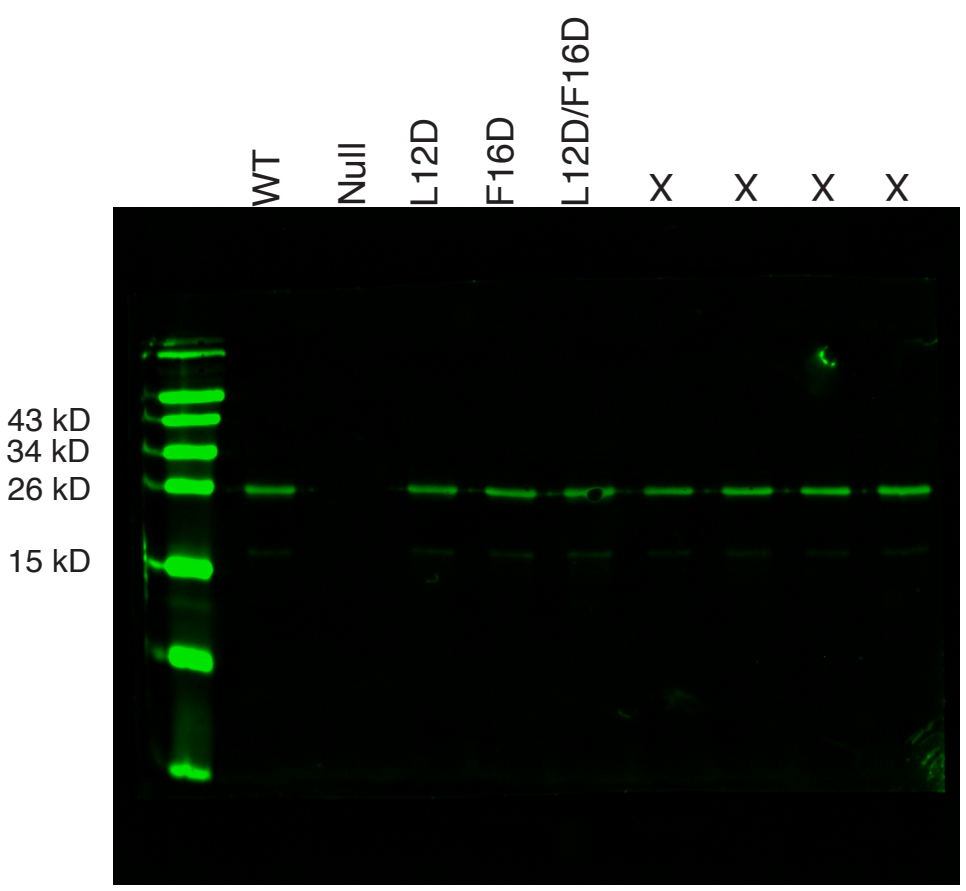

Suppl. Fig. 5d

All gel blot images were captured on an Invitrogen iBright CL1500 using fluorescent blot detection for Alexa Fluor 633.
