# Supplementary material for: The pod components of the Shigella T3SS sorting platform accommodate multiple copies of Spa33 (SctQ)
Source: PLoS Pathog. 2026 Sep 21;22(9):e1014592. doi: 10.1371/journal.ppat.1014592 (PMC13614641; doi:10.1371/journal.ppat.1014592)
Supplement: Supplementary file 2 [file ppat.1014592.s001.docx]

**Supplemental Materials for…**

**The pod components of the *Shigella* T3SS sorting platform accommodate multiple copies of Spa33 (SctQ)**

Sean K, Whittier^1,2^, Shoichi Tachiyama^3,4^, Samira Heydari^3,4^, Wendy L. Picking^1,2^, Jun Liu^3,4^ and William D. Picking^1,4^*

^1^Department of Veterinary Pathobiology, ^2^Department Christopher S. Bond Life Sciences Center, University of Missouri, Columbia, MO 65211, ^3^Department of Microbial Pathogenesis, Yale University West Haven, CT 06516, and ^4^Microbial Sciences Institute, Yale University, West Haven, CT 06516

*Corresponding author: pickingw@missouri.edu

**Key words:** *Shigella*, type III secretion system, injectisome, Spa33, SctQ, sorting platform

**Fig SA:** Canonical model of the T3SS injectisome featuring extracellular needle extending from the outer membrane (OM), periplasm spanning basal body, and sorting platform on cytoplasmic side of inner membrane (IM). Positions of SP proteins are shown, including SctD cytoplasmic domain, SctK adaptor, SctQ pod protein, and SctL spoke.

**Fig SB:** a) Overlay of single Spa33/MxiK complex (white) with single SpaO/OrgA complex (red). b) Hydrophobic surface (gold is hydrophobic, cyan hydrophilic) of MxiK at the Spa33 interaction sites. The site1 interacting region of Spa33 is shown as white cartoon. Hydrophobic patch at binding site 2 is exposed here. c) pLDDT graph for the Spa33/Spa33/MxiK complex shown in main text Figure 1a. d) Contact mediated hemolysis data for Spa33 alanine mutants. Only the F67A/F70A double mutant has significant impact on hemoylysis.

**Fig SC:** AlphaFold generated 2SctQ:SctK complexes from a) Salmonella, b) Yersinia, and c)Pseudomonas, with corresponding PAE graphs below each cartoon.

**Fig SD:** Western blots of secreted IpaB and IpaD following 20 minute incubation of Shigella flexneri strains in PBS containing Congo Red. a) Spa33 mutants, b) MxiK site 1 mutants, and c) MxiK site 2 mutants. Quantification of these data are shown in main text Figure 1, g and h.

**Fig SE:** Western blots using anti-His antibody of a) and b) Spa33-His variants; c) and d) His-MxiK variants. Null refers to uncomplemented Δspa33 or ΔmxiK cells. FL in panel (a) refers to a mutant of Spa33 in which the internal translation start site that produces Spa33_short_ has been removed. Expected sizes for epitope-tagged proteins are: Spa33-His: 39 kDa; Spa33_short_-His: 17.5 kDa; His-MxiK: 25 kDa.

**Fig SF:** Comparison of Spa33/Spa33_short_ complex (a) with SpaO/SpaO_short_ complex in (b), with corresponding structures colored according pLDDT values and PAE graphs below.

**Fig SG:** Placement of upper pod model in density map of the Shigella sorting platform with MxiK and Spa33 in positions typically described in the literature. The model does not fit the density as well as positioning shown in main text Fig. 3, with a cross-correlation value at this position of 0.41.

**Fig SH:** Three separate AlphaFold predictions for the MxiN dimer structure, colored according to pLDDT scores, with red indicating very low confidence, yellow low confidence, and dark blue very high confidence.

**Fig SI: a)** AlphaFold structure for a Spa33 dimer with individual Spa33 molecules colored white and purple. In this model, the Spa33_C_ domain of one Spa33 bind between the N- and C-terminal domains of the second Spa33 molecule in a manner nearly identical to the Spa33_short_ dimer. PAE values (bottom) for the Spa33 dimer are much higher for intermolecular regions (off-diagonal blocks) than seen in the Spa33/Spa33_short_ complex, indicating that AlphaFold is much less confident that its predicted Spa33 dimer is close to the “true” structure. **b)** AlphaFold prediction for four copies of Spa33. Here AlphaFold generates two copies of the complex shown in panel **a)**, with each dimer placed next to each other with very high PAE values for the dimer-dimer interaction (off diagonal blocks in PAE plot below). **c)** AlphaFold model of four copies of Spa33 and four copies of Spa33_short_. As indicated by off-diagonal boxes in PAE graph below, AlphaFold is not confident in interactions between the separate copies of the Spa33/Spa33_short_ complexes.

**Fig SJ:** Placement of a fourth copy of Spa33_N_ (blue) and Spa33_C_ (yellow) into the lower region of the pod. Together they fill most of the remaining density, leaving little remaining space for the two copies of MxiN.
